# Efficacy and safety of carnitine supplementation on NAFLD: a systematic review and meta-analysis

**DOI:** 10.1186/s13643-023-02238-w

**Published:** 2023-04-29

**Authors:** Aiping Liu, Yitong Cai, Yuan Yuan, Ming Liu, Zhengjing Zhang, Yongquan Xu, Pingzu Jiao

**Affiliations:** 1 School of Traditional Chinese Medicine, Gansu Health Vocational College, No. 1666 Jiulongjiang Street, Vocational Education Park, Lanzhou New District, Lanzhou City, 730314 China; 2 Xiangya School of Nursing, Central South University, Changsha City, China; 3 Gansu Provincial Central Hospital, Lanzhou City, China; 4 Gansu Provincial Maternal and Child Health Hospital, Lanzhou City, China; 5 Evidence-Based Medicine Centre, Lanzhou University, Lanzhou City, China

**Keywords:** L-carnitine, Non-alcoholic fatty liver disease, Efficacy, Systematic review, Meta-analysis

## Abstract

**Background and objective:**

The efficacy and safety of L-carnitine supplementation on non-alcoholic fatty liver disease (NAFLD) are unclear. This systematic review and meta-analysis aimed to assess the efficacy and safety of L-carnitine supplementation on NAFLD.

**Methods:**

We searched in four databases (PubMed, Embase, Cochrane Library, and Web of Science) from inception to 1 November 2022 (updated on March 20, 2023) for potentially relevant records without language restrictions. We collected information on the first author, publication year, country, setting, study design, population characteristics, duration of follow-up, outcome variables of interest, and sources of funding. We used a modified Cochrane risk of bias tool to assess the risk of bias, used GRADE to assess the certainty of evidence, and used the Credibility of Effect Modification Analyses (ICEMAN) tool to assess the credibility of any apparent subgroup effect.

**Results:**

This systematic review and meta-analysis included eight eligible randomized controlled trials (RCTs). Compared to placebo, low certainty evidence show that L-carnitine supplementation significantly changes (reduced) more in AST levels and ALT levels (MD: − 26.38, 95%CI: − 45.46 to − 7.30), and moderate certainty evidence show that L-carnitine supplementation significantly changes (reduced) more in HDL cholesterol levels (MD: 1.14, 95%CI: 0.21 to 2.07) and triglyceride levels (MD: − 6.92, 95%CI: − 13.82 to − 0.03). Moderate credibility of ICEMAN results shows that L-carnitine supplementation has no difference in changes of AST and ALT levels in younger ones (MD: 0.5, 95%CI: − 0.70 to 1.70) but has significant changes (reduced) in adults (MD: − 20.3, 95%CI: − 28.62 to − 12.28) compared to placebo.

**Conclusion:**

L-carnitine supplementation may improve liver function and regulate triglyceride metabolism in patients with NAFLD, and with no significant adverse effects.

**Supplementary Information:**

The online version contains supplementary material available at 10.1186/s13643-023-02238-w.

Non-alcoholic fatty liver disease (NAFLD) is a condition where the accumulation of lipids exceeds 5% of hepatocytes when no other causes (e.g., heavy alcohol consumption, drug consumption) [1]. There are various stages in the progression of NALFD; the initial stages of non-alcoholic fatty liver (NAFL) can progress to non-alcoholic steatohepatitis (NASH) characterized by persistent inflammatory processes, which in turn can progress to NASH fibrosis [2]. In NASH, hepatic steatosis is associated with hepatic inflammation that may be histologically indistinguishable from alcoholic steatohepatitis. Worldwide, NAFLD has a reported prevalence of 6 to 35 percent (median 20%). The pathogenesis of NAFLD has not been fully elucidated, but the most widely supported theory implicates steatohepatitis as the key mechanism leading to hepatic steatosis [3, 4]. Therefore, for NAFLD that cannot be controlled by weight loss or lifestyle interventions, any treatments that can stop hepatocellular steatosis or inflammatory lesions can be used for treatment [5–7].

Carnitine or L-b-hydroxy-c-N-trimethylaminobutyric acid is synthesized in the liver and kidneys. It was reported that carnitine can facilitates the transfer of long-chain fatty acids across the mitochondrial inner membrane as acylcarnitine esters and acts as an obligatory cofactor for β-oxidation of fatty acids [8]. Carnitine is present in almost all animal species, as well as in several microorganisms and in some higher plants [9]. L-carnitine in humans is both endogenously synthesized and obtained through food ingestion and is also used as a drug. For example, the US Food and Drug Administration (FDA) approved the use specifically of the intravenous (IV) formulation of L-carnitine in dialysis patients [10]. As an obligatory cofactor for the oxidation of fatty acids or other mechanisms, L-carnitine can be used to treat NAFLD [11]. Some evidence suggests that L-carnitine treatment for NAFDLA is effective [11, 12], but some studies have concluded the opposite [13]. These gaps suggest that a higher level of evidence is needed to address this issue [14].

A meta-analysis published in 2020 shows that L-carnitine supplementation for patients with non-alcoholic fatty liver disease demonstrates a reduction in AST, ALT, TG levels, and HOMA-IR [15]. However, this study did not use Grading of Recommendations Assessment, Development and Evaluation (GRADE) to assess the level of evidence, creating some resistance to clinical dissemination. In addition, this meta-analysis included few trials and had a low sample size and a high risk of bias. Coinciding with the publication of a new clinical trial [16–18], it is necessary to update this evidence. Therefore, this systematic review and meta-analysis aimed to assess the efficacy and safety of L-carnitine supplementation on NAFLD.

## Methods

We completed this systematic review and meta-analysis according to the Cochrane Handbook for Systematic Reviews of Interventions [19] and the GRADE guidance [20]. We reported it following the Preferred Reporting Items for Systematic Reviews and Meta-analyses (PRISMA) guidance [21]. We registered this study prospectively in Open Science Framework, https://osf.io/8xtcp.

### Search strategy and study eligibility

We searched in four databases (PubMed, Embase, Cochrane Library, and Web of Science) from inception to 1 November 2022 for potentially relevant records without language restrictions (for search terms used, see the Appendix: Text S1). The search was updated on March 20, 2023. We also hand-searched reference lists of relevant articles and searched for relevant studies from the abstract of conferences. Search strategies for all databases were completed under the guidance of a literature search specialist (JH T).

In this systematic review and meta-analysis, we considered studies eligible for inclusion if: (1) study style—randomized controlled trials (RCTs); (2) participants—patients of all ages with non-alcoholic fatty liver disease (NAFLD); (3) intervention—patients received carnitine supplement or carnitine supplement plus other nutrients; (4) comparison—patients received the same treatment as the intervention group with the exception of carnitine; and (5) outcomes—reported at least one outcome we interest, including liver function tests (AST, ALT, γ-GT), lipid profile tests (HDL cholesterol, LDL cholesterol, total cholesterol, triglyceride), body indicators (BMI, weight, waist circumference), inflammatory factors (hs-CRP), and adverse events. Conference papers are also included when data are available. Potential studies in non-English were translated with the aid of translation software or translators, if necessary.

Study selection was performed in two phases: firstly, paired reviewers independently screened the titles and abstracts; secondly, another paired reviewers independently screened the full-text review of potentially eligible records. Disagreements were resolved by consensus and, if necessary, through discussion with a third reviewer.

### Data extraction

We collected information on the first author, publication year, country, setting, study design, population characteristics, duration of follow-up, outcome variables of interest, and sources of funding. In the case of multiple records pertaining to the same trial (i.e., original full­text publication, abstract, and post hoc analyses), we collected all relevant data and analyzed them as a single study. Conversely, if a single record was reported on more than one study, we treated each study as a separate study in the analysis. Data collection was done by paired reviewers independently and checked by the other reviewer. Any disagreements were resolved by consensus and, if necessary, through discussion with a fourth reviewer.

### Risk of bias assessment

The risk of bias of the included studies was assessed independently by paired reviewers according to a modified Cochrane risk of bias tool [22]. This updated risk of bias tool consists of the 10 domains: random sequence generation; allocation concealment; blinding of participants, healthcare providers, data collectors, outcome assessors, data analysts; incomplete outcome data; selective outcome reporting; and other sources of bias (i.e., early trial discontinuation). Paired reviewers independently assessed the risk of bias and checked by the other reviewer. Any disagreements were resolved by consensus and, if necessary, through discussion with another reviewer.

### Statistical analysis

All analyses were conducted using the RStudio version 1.4.17.17 software by the packages “meta” and “metafor.” We used the method suggested by the Cochrane handbook to calculate the sample size and event [19]. We calculated the effect size as a standardized mean difference of the final scores and summarized it using a Hartung-Knapp-Sidik-Jonkman (HKSJ) random effects meta-analysis, and we switched to the DerSimonian-Laird random effects model if meta-analysis results from HKSJ were counter-intuitive. For dichotomous outcomes (adverse events), we calculated relative risks (RRs) with 95% confidence intervals (CIs). We pooled all continuous outcomes reported by more than one study as the weighted mean difference (WMD) and the associated 95%CIs.

We tested the heterogeneity of meta-analysis results by using the Cochrane *Q* test and quantified it as *I*^2^ values and the between-study variance *τ*^2^. Significance for heterogeneity was set at *p* < 0.05 and with an *I*^2^ > 50% considered to be evidence of substantial heterogeneity. We used the subgroup analyses to explore the sources of heterogeneity. When 10 or more trials were available for an outcome, we also used meta-regression to explore the sources of heterogeneity as an extension to subgroup analysis. We used the contour-enhanced funnel plots to assess publication bias, if 10 or more trials were available for an outcome (Harbord’s test for dichotomous outcomes and Egger’s test for continuous outcomes) [23, 24].

### Subgroup analysis

In order to identify the subgroup differences and potential sources of the observed heterogeneity. We performed the following prespecified subgroup analysis, if data is available. After completion, we assessed the credibility of any apparent subgroup effect using the Credibility of Effect Modification Analyses (ICEMAN) tool [25]: (1) health status—NAFLD versus NASH; hypothesis: may be no difference between the two populations; (2) age: younger ones (< 18 years) versus adult (≥ 18 years); hypothesis: no difference in the efficacy of carnitine between younger ones and adults; (3) daily dose: ≥ 1000 mg verse < 1000 mg; hypothesis: more daily doses have better efficacy and may have more adverse events; and (4) duration: 12 weeks versus 24 weeks; the longer the duration of treatment, the better the efficacy.

### Certainty of evidence

Paired reviewers with experience in using GRADE assessed the certainty of evidence independently and resolved discrepancies by discussion. We rated the certainty for each comparison and outcome as “high,” “moderate,” “low,” or “very low,” taking into consideration the risk of bias [26], inconsistency [27], imprecision [28, 29], indirectness [30], and publication bias [31]. The definition of high certainly is that we are very confident that the true effect lies close to the estimate of the effect. The definition of moderate certainly is that we are moderately confident in the effect estimate (the true effect is likely to be close to the estimate of the effect, but there is a possibility that it is substantially different). The definition of low certainty is that our confidence in the effect estimate is limited (the true effect may be substantially different from the estimate of the effect). The definition of very low certainly is that we have very little confidence in the effect estimate (the true effect is likely to be substantially different from the estimate of effect).

## Results

### Study identification

**Fig. 1:**
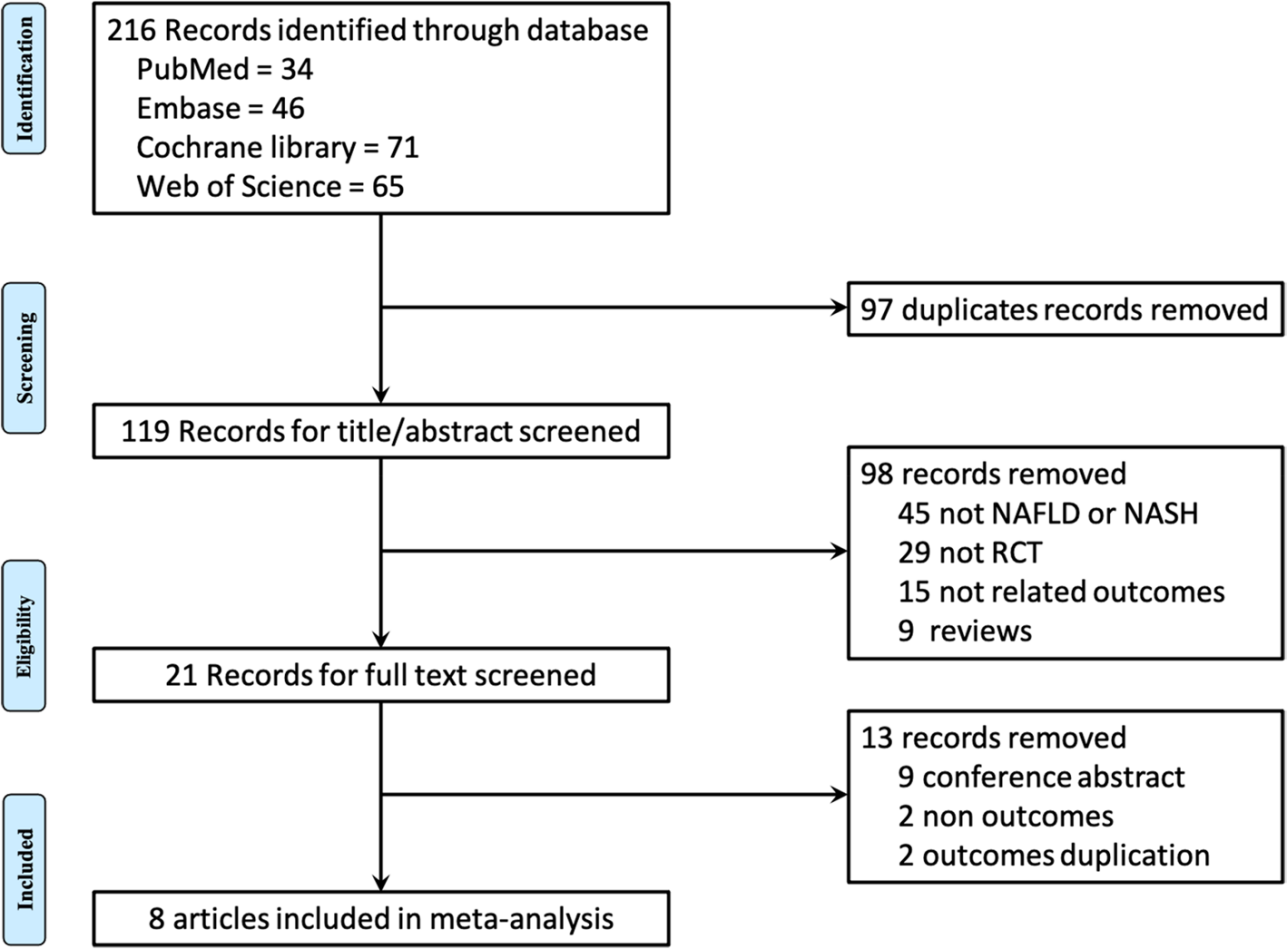
Study selection process

We initially identified 216 records from four databases, and zero studies were identified from references of relevant reviews. After removing 97 duplicates and screening 119 titles and abstracts and 9 full texts, eight eligible RCTs were included [13, 16–18, 32–35] in the final meta-analysis (Fig. 1).

### Study characteristics

Table 1 shows a summary of included eight RCTs. The eight RCTs were published between 2010 and 2021 and conducted in three countries, including Iran (*n* = 5) [13, 16–18, 35], Korea (*n* = 2) [32, 33], and Italy (*n* = 1) [34]. The patient’s health status was NAFLD in five RCTs [13, 18, 32, 33, 35] and NASH in three studies [16, 17, 34], and Appendix: Table S1 shows the inclusion and exclusion criteria of each RCT. In total, 274 participants were randomly assigned to L-carnitine or C-carnitine, and 270 were randomly assigned to placebo. The mean age of all participants (*n* = 544) ranged from 12.6 to 59.5 years, the mean BMI (kg/cm^2^) ranged from 26.5 to 31.3, and the proportion of males ranged from 34.33 to 82.5%. Six RCTs had a duration treatment of 12 weeks [13, 16–18, 32, 33], and two RCTs had a duration treatment of 24 weeks [34, 35]. Six RCTs reported liver function tests (AST, ALT, γ-GT) [13, 18, 32–35], four RCTs reported lipid profile tests (HDL cholesterol, LDL cholesterol, total cholesterol, triglyceride) [13, 32–34], six RCTs reported body indicators (BMI, weight, waist circumference) [17, 18, 32–35], two RCTs reported inflammatory factors (hs-CRP) [16, 33], and two RCTs reported adverse events [32, 33].

**Table 1:** Basic characteristics of included studies

Author, year	Country	Health status	Sample sizes (intervention/Control)	Male (*n*, %)	Age (mean year)	BMI (kg/m^2^)	Intervention	Daily dose	Control	Duration (weeks)	Outcomes
Hossein, 2021 [18]	Iran	NAFLD	31/31	41, 66.13%	12.6	26.7	L-carnitine	1000 mg	Placebo	12	①③⑤
Shirin, 2015 (1) [17]	Iran	NASH	36/35	23, 34.33%	43.3	31.3	L-carnitine	2000 mg	Placebo	12	③
Shirin, 2015 (2) [16]	Iran	NASH	36/35	23, 34.33%	43.3	31.3	L-carnitine	2000 mg	Placebo	12	④
Pezhman, 2016 [13]	Iran	NAFLD	30/30	38, 63.33%	59.5	29.1	L-carnitine	2250 mg	Placebo	12	①②
Bae, 2015 [32]	Korea	NAFLD	39/39	54, 69.23%	51.3	27.5	C-carnitine	900 mg	Placebo	12	①②③⑤
Hong, 2014 [33]	Korea	NAFLD	26/26	36, 69.23%	51.8	27.1	C-carnitine	900 mg	Placebo	12	①②③④⑤
Mariano, 2010 [34]	Italy	NASH	36/38	40, 54.05%	47.8	26.5	L-carnitine	2000 mg	Placebo	24	①②③
Mohamad, 2014 [35]	Iran	NAFLD	40/40	66, 82.50%	40.7	29.0	L-carnitine	500 mg	Placebo	24	①③

*NAFLD* non-alcoholic fatty liver disease, *NASH* non-alcoholic steatohepatitis, *C-carnitine* carnitine-orotate complex

① Liver function tests (AST, ALT, γ-GT); ② lipid profile tests (HDL cholesterol, LDL cholesterol, total cholesterol, triglyceride); ③ body indicators (BMI, weight, waist circumference); ④ inflammatory factors (hs-CRP); and ⑤ adverse events

### Quality assessment

Appendix: Table S2 presents the risk of bias of included RCTs for each outcome. One study [35] was at the “definitely low” or “probably low” in all domains. Three studies [32–34] were at the “definitely low” or “probably low” or “probably high” in all domains. All outcomes of the four studies [13, 16–18] were “high” in the domain of “incomplete outcome data.”

### Meta-analysis outcomes

#### Liver function tests

**Fig. 2:**
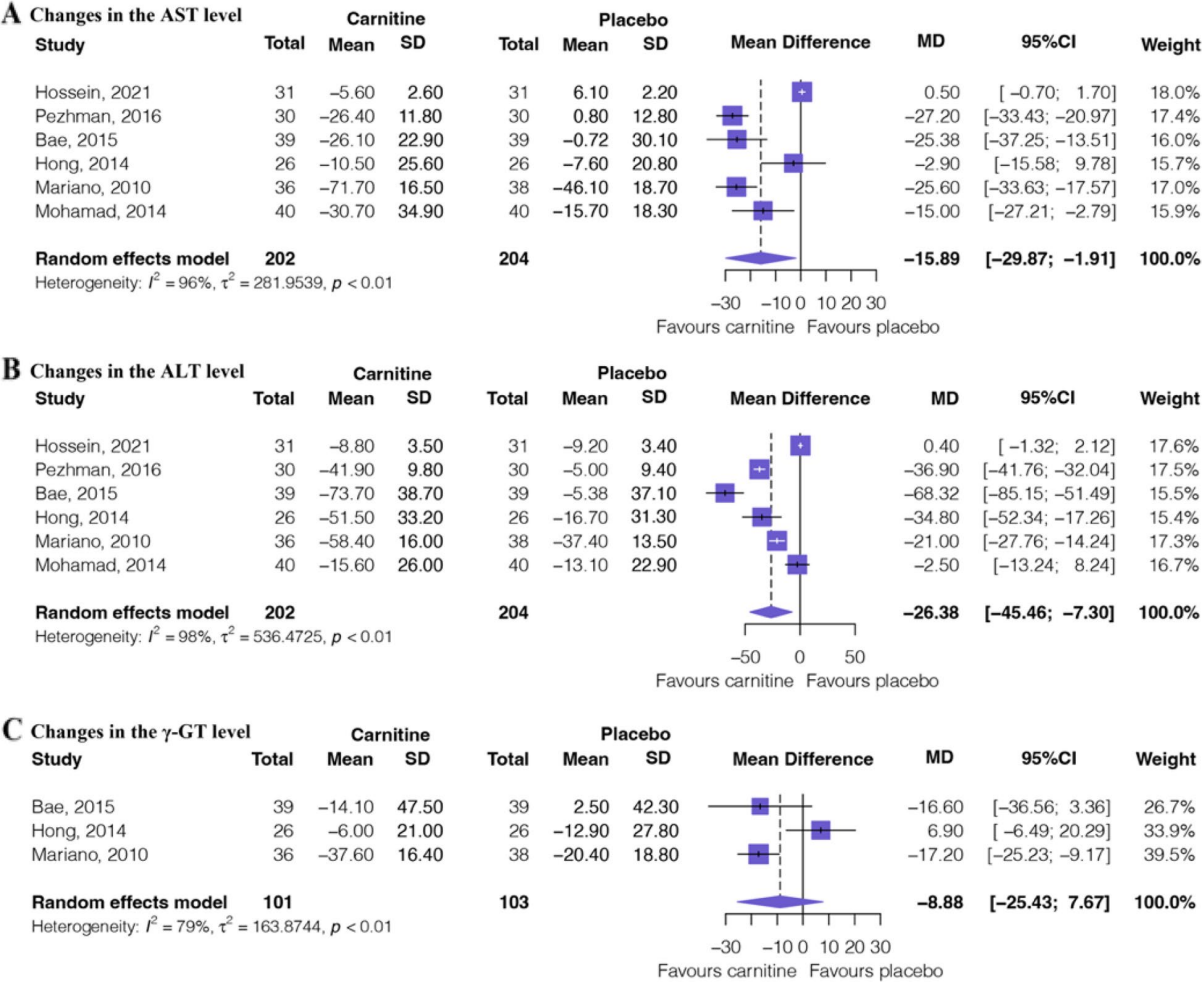
Forest plot of liver function texts. **A** Changes in the AST levels. **B** Changes in the ALT levels. **C** Changes in the γ-GT levels

Figure 2 shows the outcomes of the meta-analysis of liver function texts. Six RCTs [13, 18, 32–35] involving 406 patients provided low certainty evidence (Table 2) that L-carnitine supplementation significantly changes (reduced) in the AST levels (MD: − 15.89, 95%CI: − 29.87 to − 1.91) and low certainty evidence (Table 2) that L-carnitine supplementation significantly changes (reduced) in the ALT levels (MD: − 26.38, 95%CI: − 45.46 to − 7.30). Three RCTs [32–34] involving 204 patients provided low certainty evidence (Table 2) that L-carnitine supplementation may induce or no difference in changes in the γ-GT levels (MD: − 8.88, 95%CI: − 25.43 to 7.67) [34].

**Table 2:** Certainty of evidence of each outcome

Outcome	Study results and measurements	Absolute effect estimates		Certainty of the evidence	Plain language summary
		**Placebo**	**L-carnitine**		
AEs	Relative risk: 0.72 (CI 95% 0.47–1.08) Based on data from 192 participants in 3 studies Follow-up 12 weeks	**323** per 1000	**233** per 1000	**Moderate**^a^	L-carnitine probably has little or no difference on AEs
		Difference: **90 fewer per 1000** (CI 95% 171 fewer to 26 more)			
Changes in the AST level	Measured by: Scale: high better Based on data from 406 participants in 6 studies Follow-up 12/24 weeks	**6.85**		**Low**^b^	L-carnitine may increase changes in the AST level
		Mean	Mean		
		Difference: **MD 15.89 lower** (CI 95% 29.87 lower to 1.91 lower)			
Changes in the ALT level	Measured by: Scale: high better Based on data from 406 participants in 6 studies Follow-up 12/24 weeks	**11.15**		**Low**^b^	L-carnitine may increase changes in the ALT level
		Mean	Mean		
		Difference: **MD 26.38 lower** (CI 95% 45.46 lower to 7.30 lower)			
Changes in the γ-GT level	Measured by: Scale: high better Based on data from 204 participants in 3 studies Follow-up 12/24 weeks	**12.9**		**Low**^b^	L-carnitine may have little or no difference in changes in the γ-GT level
		Mean	Mean		
		Difference: **MD 8.88 lower** (CI 95% 25.43 lower to 7.67 lower)			
Changes in the HDL cholesterol level	Measured by: Scale: high better Based on data from 204 participants in 3 studies Follow-up 12/24 weeks	**0.5**		**Moderate**^a^	L-carnitine probably increases changes in the HDL cholesterol level
		Mean	Mean		
		Difference: **MD 1.14 higher** (CI 95% 0.21 higher to 2.07 higher)			
Changes in the LDL cholesterol level	Measured by: Scale: high better Based on data from 204 participants in 3 studies Follow-up 12/24 weeks	**5.4**		**Low**^b^	L-carnitine may have little or no difference in changes in the LDL cholesterol level
		Mean	Mean		
		Difference: **MD 6.8 lower** (CI 95% 23.27 lower to 9.68 higher)			
Changes in the total cholesterol level	Measured by: Scale: high better Based on data from 186 participants in 3 studies Follow-up 12/24 weeks	**6**		**Low**^b^	L-carnitine may have little or no difference in changes in the Total cholesterol level
		Mean	Mean		
		Difference: **MD 11.8 lower** (CI 95% 27.13 lower to 3.53 higher)			
Changes in the triglyceride level	Measured by: Scale: high better Based on data from 264 participants in 4 studies Follow-up 12/24 weeks	**6.24**		**Moderate**^a^	L-carnitine probably increases changes in the triglyceride level
		Mean	Mean		
		Difference: **MD 6.92 lower** (CI 95% 13.82 lower to 0.07 lower)			
Changes in the BMI	Measured by: Scale: high better Based on data from 417 participants in 6 studies Follow-up 12/24 weeks	**0.45**		**Moderate**^a^	L-carnitine probably has little or no difference in changes in the BMI
		Mean	Mean		
		Difference: **MD 0 lower** (CI 95% 0.23 lower to 0.24 higher)			
Changes in the waist circumference	Measured by: Scale: high better Based on data from 211 participants in 3 studies Follow-up 12 weeks	**1.5**		**Low**^b^	L-carnitine may have little or no difference in changes in the waist circumference
		Mean	Mean		
		Difference: **MD 0.57 lower** (CI 95% 1.82 lower to 0.67 higher)			
Changes in the weight	Measured by: Scale: high better Based on data from 291 participants in 4 studies Follow-up 12/24 weeks	**0.6**		**Moderate**^a^	L-carnitine probably has little or no difference in changes in the weight
		Mean	Mean		
		Difference: **MD 0.2 lower** (CI 95% 0.5 lower to 0.09 higher)			
Changes in the hs-CRP	Measured by: Scale: high better Based on data from 123 participants in 2 studies Follow-up 12 weeks	**0.27**		**Very low**^c^	We are uncertain whether L-carnitine increases or decreases changes in the hs-CRP
		Mean	Mean		
		Difference: **MD 1.03 lower** (CI 95% 3.23 lower to 1.16 higher)			

^a^Rated down 1 level for risk of bias due to incomplete data

^b^Rated down 2 levels for risk of bias due to incomplete data and for serious inconsistency due to statistical heterogeneity

^c^Rated down 2 levels for risk of bias due to incomplete data, for serious inconsistency due to statistical heterogeneity, and for serious imprecision due to few patients

#### Lipid profile tests

**Fig. 3:**
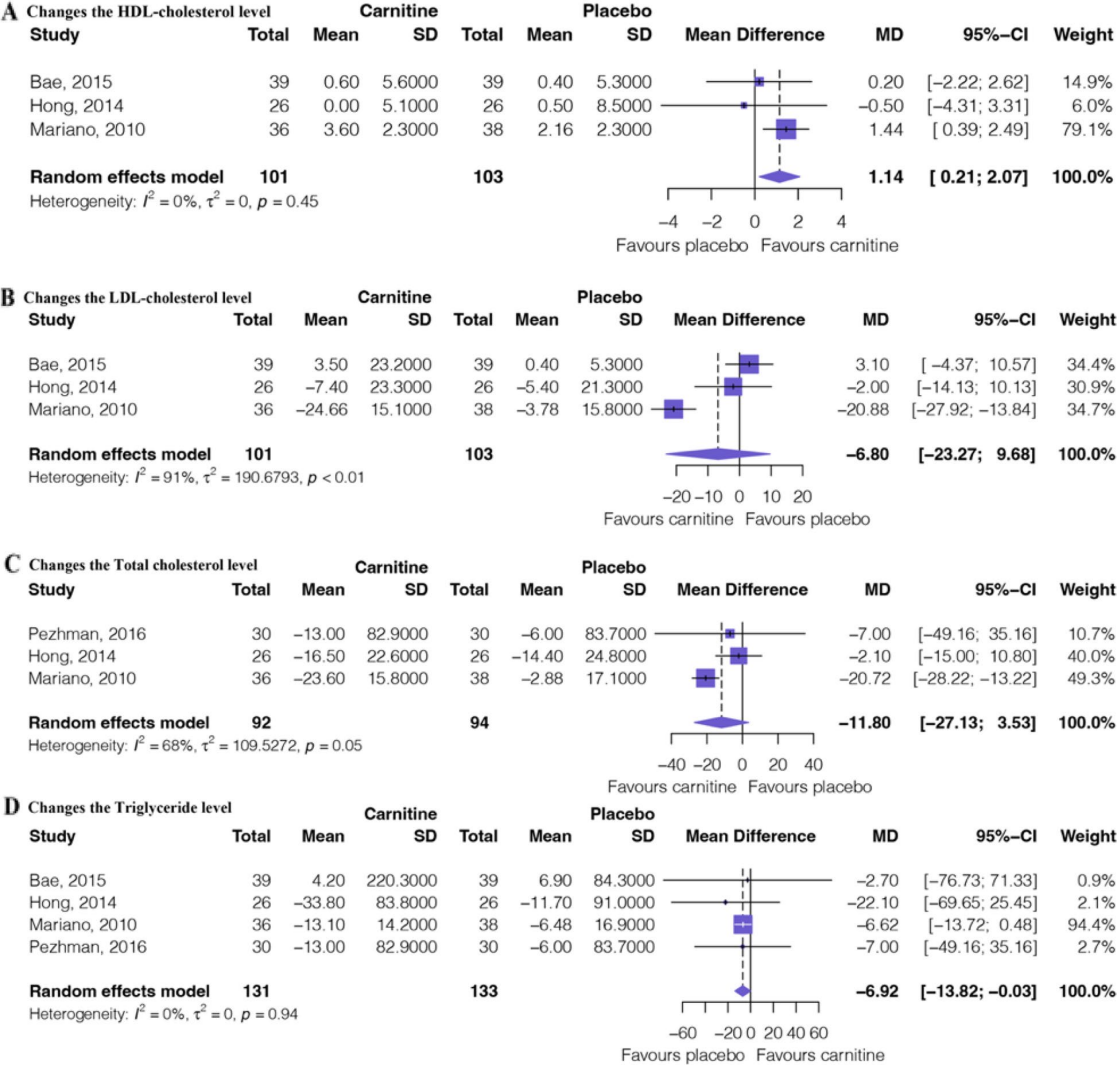
Forest plot of lipid profile texts. **A** Changes in the HDL cholesterol levels. **B** Changes in the LDL cholesterol levels. **C** Changes in the triglyceride levels. **D** Changes in the total cholesterol levels

Figure 3 shows the outcomes of the meta-analysis of lipid profile texts. Three RCTs [32–34] involving 204 patients provided moderate certainty evidence (Table 2) that L-carnitine supplementation significantly changes in the HDL cholesterol levels (MD: 1.14, 95%CI: 0.21 to 2.07) and low certainty evidence (Table 2) that L-carnitine supplementation may induce or no difference on changes in the LDL cholesterol levels (MD: − 6.80, 95%CI: − 23.27 to 9.68). Three RCTs [13, 33, 34] involving 186 patients provided low certainty evidence (Table 2) that L-carnitine supplementation may induce or no difference in changes in the total cholesterol levels (MD: − 11.80, 95%CI: − 27.13 to 3.53). Four RCTs [13, 32–34] involving 264 patients provided moderate certainty evidence (Table 2) that L-carnitine supplementation may induce the triglyceride levels (MD: − 6.92, 95%CI: − 13.82 to − 0.03).

#### Body indicators

**Fig. 4:**
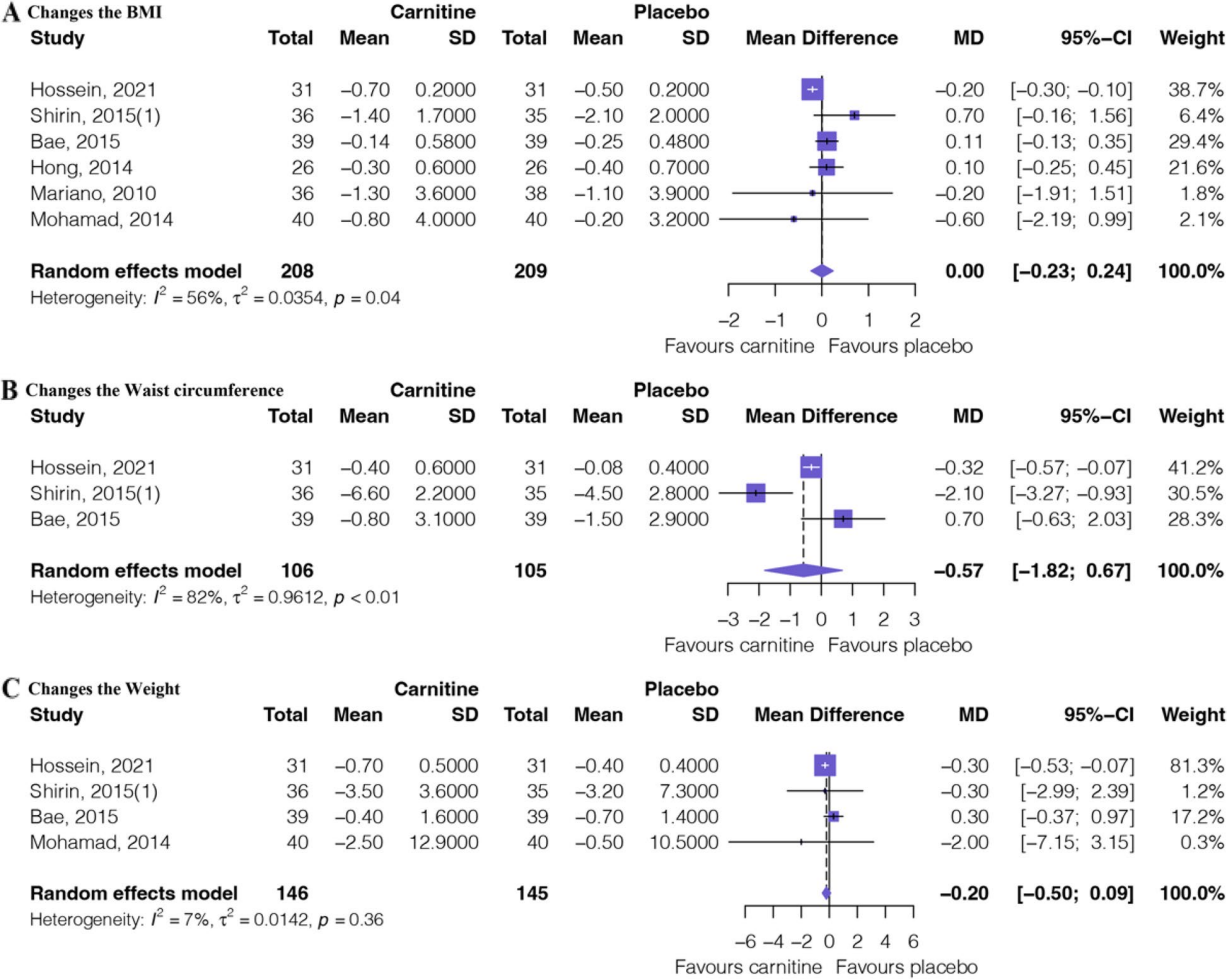
Forest plot of lipid profile texts. **A** Changes in the BMI. **B** Changes in the waist circumference. **C** Changes in the weight

Figure 4 shows the outcomes of the meta-analysis of body indicators. Six RCTs [17, 18, 32–35] involving 417 patients provided moderate certainty evidence (Table 2) that L-carnitine supplementation has no difference in changes in the BMI (MD: 0.00, 95%CI: − 0.23 to 0.24). Three RCTs [17, 18, 32] involving 211 patients provided low certainty evidence (Table 2) that L-carnitine supplementation has no difference in changes in waist circumference (MD: − 0.57, 95%CI: − 1.82 to 0.67). Four RCTs [17, 18, 32, 35] involving 291 patients provided moderate certainty evidence (Table 2) that L-carnitine supplementation has no difference in changes in weight (MD: − 0.20, 95%CI: − 0.50 to 0.09).

#### Inflammatory factor

**Fig. 5:**
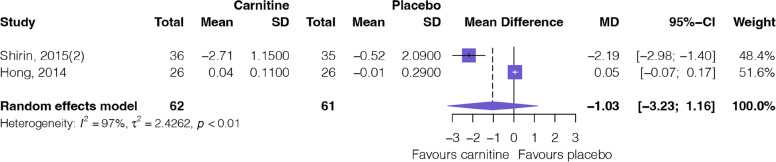
Forest plot of inflammatory factor (hs-CRP)

Figure 5 shows the outcomes of the meta-analysis of inflammatory factors. Two RCTs [16, 33] involving 123 patients provided very low certainty evidence (Table 2) that L-carnitine supplementation has no difference in changes in the hs-CRP (MD: − 1.03, 95%CI: − 3.23 to 1.16).

#### Safety

**Fig. 6:**
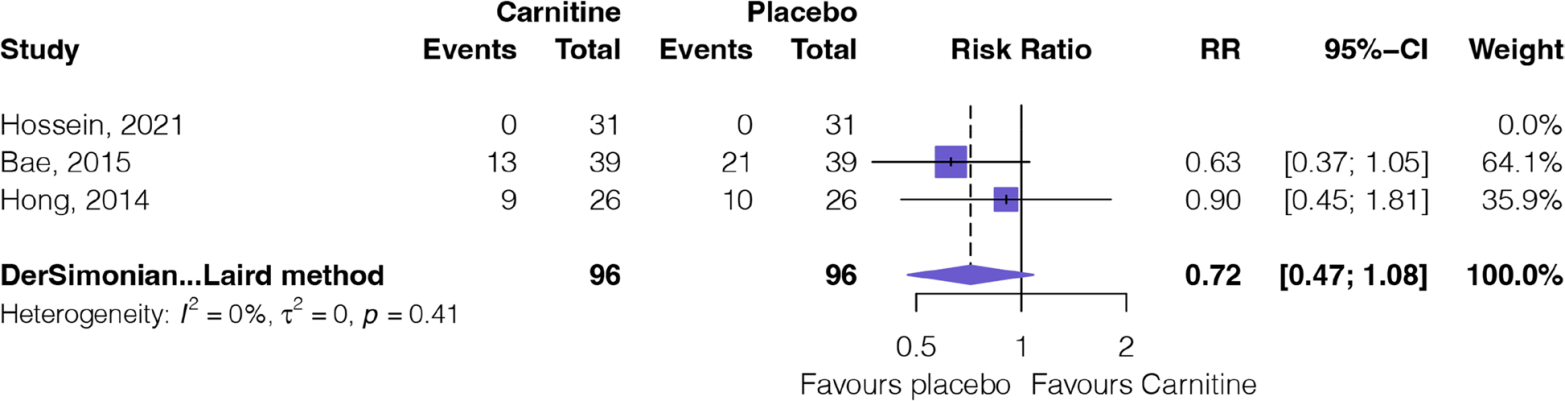
Forest plot of adverse events

Figure 6 shows the outcomes of the meta-analysis of adverse events. Three RCTs [18, 32, 33] involving 192 patients provided moderate certainty evidence (Table 2) that L-carnitine supplementation probably has little or no difference in adverse events (RR: 0.72, 95%CI: 0.47 to 1.08).

#### Subgroup analysis and other analysis

Appendix: Table S3 shows the subgroup analysis results. Subgroup analyses identified no suggestion of subgroup effects for the outcome of changes in the γ-GT, HDL cholesterol, and triglyceride level, and changes in the weight and waist circumference. Therefore, the use of ICEMAN to assess the subgroup effects of the above outcomes was not applicable. In the outcome of changes in the AST levels, moderate credibility of ICEMAN result shows that L-carnitine supplementation has no difference in changes in younger ones (MD: 0.5, 95%CI: − 0.70 to 1.70) but have significant changes (reduced) in adults (MD: − 20.3, 95%CI: − 28.62 to − 12.28). In the outcome of changes in the ALT levels, moderate credibility of ICEMAN result shows that L-carnitine supplementation has no difference in changes in younger ones (MD: 0.4, 95%CI: − 1.32 to 2.12) but has significant changes (reduced) in adults (MD: − 31.7, 95%CI: − 47.61 to − 15.79).

In the outcome of changes in the LDL cholesterol levels, low credibility of ICEMAN result shows that carnitine supplementation has no difference in changes in NAFLD/daily dose ≥ 1000 mg/duration_12 weeks (MD: 1.7, 95%CI: − 4.66 to 8.06) but has significant changes (reduced) in the NASH/daily dose < 1000 mg/duration_24 weeks (MD: − 20.9, 95%CI: − 27.92 to − 13.84). In the outcome of changes in the total cholesterol levels, low credibility of ICEMAN result shows that L-carnitine supplementation has no difference in changes in NAFLD/duration_12 weeks (MD: − 2.5, 95%CI: − 14.85 to 9.81) but has significant changes (reduced) in the NASH/duration_24 weeks (MD: − 20.7, 95%CI: − 28.22 to − 13.22); low credibility of ICEMAN result shows that carnitine supplementation has no difference in changes in daily dose < 1000 mg (MD: − 2.1, 95%CI: − 15.00 to 10.80) but has significant changes (reduced) in the daily dose ≥ 1000 mg (MD: − 20.3, 95%CI: − 27.68 to − 12.92).

In the outcome of changes in the BMI, moderate credibility of ICEMAN result shows that L-carnitine supplementation has no difference in changes in adults (MD: 0.1, 95%CI: − 0.07 to 0.31) but has significant changes (reduced) in the younger ones (MD: − 0.2, 95%CI: − 0.30 to − 0.10). In the outcome of changes in the hsCRP, very low credibility of ICEMAN result shows that carnitine supplementation has no difference in changes in daily dose < 1000 mg (MD: 0.05, 95%CI: − 0.07 to 0.17) but has significant changes (reduced) in the daily dose ≥ 1000 mg (MD: − 2.19, 95%CI: − 2.98 to − 1.40).

In addition, sensitivity analyses after excluding zero-event studies showed similar results to the primary analyses. We did not evaluate the publication bias because none of the outcomes included more than 10 studies.

## Discussion

We found low certainly evidence that L-carnitine supplementation significantly reduce the AST/ALT levels for patients with NAFLD compared to placebo. Analysis showed that L-carnitine reduced the AST by a mean of 6.85 IU/L when used for the treatment of NAFLD and reduced the ALT by a mean of 11.15 IU/L. However, moderate credibility subgroup analysis showed that L-carnitine was effective to reduce AST/ALT levels in adults but failed in younger ones. Moderate certainty evidence showed that L-carnitine supplementation has significant changes in the HDL cholesterol and triglyceride levels for patients with NAFLD compared to placebo. Analysis showed that L-carnitine improved the HDL cholesterol by a mean of 0.5 mmol/l when used for the treatment of NAFLD and reduced the triglyceride by a mean of 6.24 mg/ml.

### Strengths and weaknesses of this review

This systematic review and meta-analysis was registered and reported with PRISMA [21] and included the full of original trials whenever possible. The review included more studies than previous reviews and covered more outcomes. The heterogeneity in this study was substantial, and we speculate that most of the heterogeneity may have come from clinical sources. Therefore, the inclusion–exclusion criteria for each original study are provided in the Appendix of this study. In order to assess the study level risk of bias, we used the modified Cochrane risk of bias tool [22], and we evaluated the certainty of the evidence using the GRADE [20]. We performed a predetermined subgroup analysis and used the ICEMAN tool [25] assessing the credibility of the subgroup effect. To facilitate the interpretation of results, we presented absolute effects for all outcomes.

There are also some weaknesses in our study. Compared with the previous systematic review, although more trials and sample sizes were included in our study, it is still relatively small. Although we performed comprehensive literature searches and no restrictions on language, however, trials published in other languages may also be missed. There are two RCTs [32, 33] in which the intervention is carnitine-orotate complex, although we only counted the daily dose of L-carnitine, it is possible that other ingredients in the carnitine-orotate complex may have an impact on efficacy and safety. After all, biphenyl dimethyl dicarboxylate [36], adenine [37], cyanocobalamin [38], pyridoxine, and riboflavin may all have effects on NAFLD. In addition, the inclusion of patients with different diagnostic criteria may introduce clinical heterogeneity, and evidence users should consider this.

### Evidence update

The most comparable to our study is another meta-analysis published by Abolfathi et al. in 2020 [15]. Firstly, our study had 204 patients in three more RCTs than the previous meta-analysis. More studies and patients can make the results of the analysis more reliable. Secondly, our study used the GRADE tool to assess the quality of evidence, which is of greater relevance to clinical practice. Thirdly, similar to our study, Abolfathi et al. also found that L-carnitine can improve liver function in patients with NAFLD. However, there are indeed different findings in improving lipids in patients with NAFLD. Our study found that L-carnitine improved HDL cholesterol levels. Finally, the present study found, by assessing the reliability of subgroup analyses, that almost all subgroup analyses were unreliable, limited by the fact that both the number of included studies and the sample size were small. They should be interpreted with caution in clinical application.

### Meaning of the study

L-carnitine is water-soluble that can be obtained not only from diet (around 75%) [39], but also synthesized in the body. It is can synthesize from the two amino acids, lysine and methionine. The key enzyme for the synthesis of L-carnitine, however, is located in the liver, kidneys, and brain [11]. If the liver is metabolically compromised, then the synthesis pathway may be inhibited. The main drivers in NAFLD are inflammation and accumulation of lipids [40], and L-carnitine has been shown to have anti-inflammatory effects by upregulating the peroxisome proliferator activator receptor-γ (PPAR-γ) in the liver [41]. L-carnitine is also closely related to fat metabolism [9]. It can be seen that there is also an interaction between abnormal liver function and the absorption of L-carnitine. The pooled analysis of clinical data in our study suggests that L-carnitine does improve liver function and affects triglyceride metabolism in patients with NAFLD. Although the level of evidence is not very high, it is the best summary of the current evidence. The results of the basic study and the clinical trials corroborate each other, which is an important significance of our study.

### Implications for clinical practice

This systematic review of eight RCTs provides detailed information for decision-makers about the benefits and harms of L-carnitine supplementation on NAFLD. Despite results from a low-confidence subgroup analysis, higher doses and longer courses of treatment may yield greater benefits without causing serious side effects. Therefore, for patients with NAFLD, especially those with a restricted diet (e.g., meat) [42], it is safe to supplement with L-carnitine. However, it is important to note in clinical practice that this does not mean that L-carnitine is a substitute for other lipid-regulating and anti-inflammatory drugs.

### Unanswered questions and future research

Although the present study provides the best evidence available, the total number of studies and sample size are inadequate. Therefore, a larger RCT is highly warranted, especially in the field of dose and duration. In addition, L-carnitine is being used as a supplementation therapy, and the best and safe combination with which therapy is used is a point to be considered in the future. Because the absorption of L-carnitine is also very critical, which is also related to liver function. The region where the patient is located is also important because the diet of people in different regions is not consistent. If possible, multicenter clinical trials should be conducted in as many regions as possible. In addition, since the concept of NAFLD is replaced by metabolic-associated fatty liver disease (MAFLD), the outcomes of our study are also applicable to MAFLD patients. Of course, further studies are needed for validation. Because some metabolic co-morbidities may affect the efficacy of L-carnitine, clinicians should evaluate patients thoroughly at the time of use. The outcomes of this study should be used with caution if patients have co-morbidities that may affect efficacy.

## Conclusion

L-carnitine supplementation may improve liver function and regulates triglyceride metabolism in patients with NAFLD, with no significant adverse effects. Multicenter and large sample clinical trials should be conducted in as many regions as possible, to explore which interventions work best when combined with L-carnitine.

## Supplementary Information

**Additional file 1:** **Text S1.** Search strategy in PubMed database. **Table S1.** Inclusion and exclusion criteria of each randomized controlled trials. **Table S2.** Risk of bias of included randomized controlled trials for each outcomes. **Table S3.** Subgroup analysis results

## References

[1] Neuschwander-Tetri BA (2017). Non-alcoholic fatty liver disease. BMC Med.

[2] Wiering L, Tacke F: Treating inflammation to combat non-alcoholic fatty liver disease. J Endocrinol 2023;256. 10.1530/JOE-22-0194 36259984

[3] Nobili V, Alisi A, Valenti L, Miele L, Feldstein AE, Alkhouri N (2019). NAFLD in children: new genes, new diagnostic modalities and new drugs. Nat Rev Gastroenterol Hepatol.

[4] Dongiovanni P, Stender S, Pietrelli A, Mancina RM, Cespiati A, Petta S, Pelusi S, Pingitore P, Badiali S, Maggioni M (2018). Causal relationship of hepatic fat with liver damage and insulin resistance in nonalcoholic fatty liver. J Intern Med.

[5] Powell EE, Wong VW, Rinella M (2021). Non-alcoholic fatty liver disease. Lancet.

[6] Singh S, Osna NA, Kharbanda KK (2017). Treatment options for alcoholic and non-alcoholic fatty liver disease: a review. World J Gastroenterol.

[7] Younossi ZM (2019). Non-alcoholic fatty liver disease - a global public health perspective. J Hepatol.

[8] Torgerson JS, Hauptman J, Boldrin MN, Sjöström L (2004). XENical in the prevention of diabetes in obese subjects (XENDOS) study: a randomized study of orlistat as an adjunct to lifestyle changes for the prevention of type 2 diabetes in obese patients. Diabetes Care.

[9] Pekala J, Patkowska-Sokoła B, Bodkowski R, Jamroz D, Nowakowski P, Lochyński S, Librowski T (2011). l-carnitine–metabolic functions and meaning in humans life. Curr Drug Metab.

[10] Nishioka N, Luo Y, Taniguchi T, Ohnishi T, Kimachi M, Ng RC, Watanabe N (2022). Carnitine supplements for people with chronic kidney disease requiring dialysis. Cochrane Database Syst Rev..

[11] Savic D, Hodson L, Neubauer S, Pavlides M. The importance of the fatty acid transporter l-carnitine in non-alcoholic fatty liver disease (NAFLD). Nutrients. 2020;12(8):2178. 10.3390/nu12082178 PMC7469009 32708036

[12] Li N, Zhao H (2021). Role of carnitine in non-alcoholic fatty liver disease and other related diseases: an update. Front Med (Lausanne).

[13] Alavinejad P, Zakerkish M, Hajiani E, Hashemi SJ, Chobineh M, Moghaddam EK (2016). Evaluation of l-carnitine efficacy in the treatment of non-alcoholic fatty liver disease among diabetic patients: a randomized double blind pilot study. J Gastroenterol Hepatol Res.

[14] Tian J, Gao Y, Zhang J, Yang Z, Dong S, Zhang T, Sun F, Wu S, Wu J, Wang J (2021). Progress and challenges of network meta-analysis. J Evid Based Med.

[15] Abolfathi M, Mohd-Yusof BN, Hanipah ZN, MohdRedzwan S, Yusof LM, Khosroshahi MZ (2020). The effects of carnitine supplementation on clinical characteristics of patients with non-alcoholic fatty liver disease: a systematic review and meta-analysis of randomized controlled trials. Complement Ther Med.

[16] Amiri-Moghadam S, Nematy M, Eghtesadi S, Khalili M, Mojarrad M, Jazayeri S, Vosooghinia H, Khosravi A, Salehi M (2015). Effects of l-carnitine supplementation on inflammatory factors and malondialdehyde in patients with nonalcoholic steatohepatitis (NASH). Curr Topics Nutraceutical Res.

[17] Amiri-Moghadam S, Nematy M, Eghtesadi S, Khalili M, Mojarrad M, Jazayeri S, Vosooghinia H, Khosravi A, Salehi M, Beheshti-Namdar A (2015). Effects of l-carnitine supplementation on body composition in patients with nonalcoholic steatohepatitis (NASH). Curr Topics Nutraceutical Res.

[18] Saneian H, Khalilian L, Heidari-Beni M, Khademian M, Famouri F, Nasri P, Hassanzadeh A, Kelishadi R (2021). Effect of l-carnitine supplementation on children and adolescents with nonalcoholic fatty liver disease (NAFLD): a randomized, triple-blind, placebo-controlled clinical trial. J Pediatr Endocrinol Metab.

[19] Cumpston MS, McKenzie JE, Welch VA, Brennan SE (2022). Strengthening systematic reviews in public health: guidance in the Cochrane Handbook for Systematic Reviews of Interventions, 2nd edition. J Public Health (Oxf).

[20] Chu DK, Golden DBK, Guyatt GH (2021). Translating evidence to optimize patient care using GRADE. J Allergy Clin Immunol Pract.

[21] Page MJ, McKenzie JE, Bossuyt PM, Boutron I, Hoffmann TC, Mulrow CD, Shamseer L, Tetzlaff JM, Akl EA, Brennan SE (2021). The PRISMA 2020 statement: an updated guideline for reporting systematic reviews. BMJ.

[22] Guyatt GH, Busse JW (2022). Modification of cochrane tool to assess risk of bias in randomized trials.

[23] Harbord RM, Egger M, Sterne JA (2006). A modified test for small-study effects in meta-analyses of controlled trials with binary endpoints. Stat Med.

[24] Egger M, Davey Smith G, Schneider M, Minder C (1997). Bias in meta-analysis detected by a simple, graphical test. BMJ.

[25] Schandelmaier S, Briel M, Varadhan R, Schmid CH, Devasenapathy N, Hayward RA, Gagnier J, Borenstein M, van der Heijden G, Dahabreh IJ (2020). Development of the Instrument to assess the Credibility of Effect Modification Analyses (ICEMAN) in randomized controlled trials and meta-analyses. CMAJ.

[26] Guyatt GH, Oxman AD, Vist G, Kunz R, Brozek J, Alonso-Coello P, Montori V, Akl EA, Djulbegovic B, Falck-Ytter Y (2011). GRADE guidelines: 4. Rating the quality of evidence–study limitations (risk of bias). J Clin Epidemiol.

[27] Perleth M, Langer G, Meerpohl JJ, Gartlehner G, Kaminski-Hartenthaler A, Schünemann HJ (2012). GRADE guidelines: 7. Rating the quality of evidence - inconsistency. Z Evid Fortbild Qual Gesundhwes.

[28] Guyatt GH, Oxman AD, Kunz R, Brozek J, Alonso-Coello P, Rind D, Devereaux PJ, Montori VM, Freyschuss B, Vist G (2011). GRADE guidelines 6. Rating the quality of evidence–imprecision. J Clin Epidemiol.

[29] Shao SC, Kuo LT, Huang YT, Lai PC, Chi CC: Using Grading of Recommendations Assessment, Development, and Evaluation (GRADE) to rate the certainty of evidence of study outcomes from systematic reviews: a quick tutorial. Dermatologica Sinica 2023, [Epub ahead of print]:[cited 2023 Mar 2021] Available from: https://www.dermsinica.org/preprintarticle.asp?id=368303.

[30] Guyatt GH, Oxman AD, Kunz R, Woodcock J, Brozek J, Helfand M, Alonso-Coello P, Falck-Ytter Y, Jaeschke R, Vist G (2011). GRADE guidelines: 8. Rating the quality of evidence–indirectness. J Clin Epidemiol.

[31] Guyatt GH, Oxman AD, Montori V, Vist G, Kunz R, Brozek J, Alonso-Coello P, Djulbegovic B, Atkins D, Falck-Ytter Y (2011). GRADE guidelines: 5. Rating the quality of evidence–publication bias. J Clin Epidemiol.

[32] Bae JC, Lee WY, Yoon KH, Park JY, Son HS, Han KA, Lee KW, Woo JT, Ju YC, Lee WJ (2015). Improvement of nonalcoholic fatty liver disease with carnitine-orotate complex in type 2 diabetes (CORONA): a randomized controlled trial. Diabetes Care.

[33] Hong ES, Kim EK, Kang SM, Khang AR, Choi SH, Park KS, Jang HC, Lim S (2014). Effect of carnitine-orotate complex on glucose metabolism and fatty liver: a double-blind, placebo-controlled study. J Gastroenterol Hepatol.

[34] Malaguarnera M, Gargante MP, Russo C, Antic T, Vacante M, Malaguarnera M, Avitabile T, Li Volti G, Galvano F (2010). l-carnitine supplementation to diet: a new tool in treatment of nonalcoholic steatohepatitis–a randomized and controlled clinical trial. Am J Gastroenterol.

[35] Somi MH, Fatahi E, Panahi J, Havasian MR, Judaki A (2014). Data from a randomized and controlled trial of Lcarnitine prescription for the treatment for non-alcoholic fatty liver disease. Bioinformation.

[36] Heo NY, Park SH, Choi JH, Kim E, Kim TO, Park J, Lee J, Park YE, Oh EH, Hwang JS, Jeong SJ (2021). Efficacy and safety of biphenyl dimethyl dicarboxylate and ursodeoxycholic acid combination in chronic hepatitis related to metabolic syndrome components. Korean J Gastroenterol.

[37] Mukherjee R, Moreno-Fernandez ME, Giles DA, Cappelletti M, Stankiewicz TE, Chan CC, Divanovic S (2018). Nicotinamide adenine dinucleotide phosphate (reduced) oxidase 2 modulates inflammatory vigor during nonalcoholic fatty liver disease progression in mice. Hepatol Commun.

[38] Spence JD (2023). B vitamins for NASH: use methylcobalamin, not cyanocobalamin. J Hepatol.

[39] Stephens FB, Marimuthu K, Cheng Y, Patel N, Constantin D, Simpson EJ, Greenhaff PL (2011). Vegetarians have a reduced skeletal muscle carnitine transport capacity. Am J Clin Nutr.

[40] El-Sheikh AA, Rifaai RA (2014). Peroxisome proliferator activator receptor (PPAR)-γ ligand, but not PPAR-α, ameliorates cyclophosphamide-induced oxidative stress and inflammation in rat liver. PPAR Res.

[41] Gruben N, Shiri-Sverdlov R, Koonen DP, Hofker MH (2014). Nonalcoholic fatty liver disease: a main driver of insulin resistance or a dangerous liaison?. Biochim Biophys Acta.

[42] Faris M, Jahrami H, Abdelrahim D, Bragazzi N, BaHammam A (2021). The effects of Ramadan intermittent fasting on liver function in healthy adults: a systematic review, meta-analysis, and meta-regression. Diabetes Res Clin Pract.

