# Supplementary material for: Efficacy and safety of carnitine supplementation on NAFLD: a systematic review and meta-analysis
Source: Syst Rev. 2023 Apr 29;12:74. doi: 10.1186/s13643-023-02238-w (PMC10148537; doi:10.1186/s13643-023-02238-w)
Supplement: Supplementary file 1 — Additional file 1: Text S1. Search strategy in PubMed database. Table S1. Inclusion and exclusion criteria of each randomized controlled trials. Table S2. Risk of bias of included randomized controlled trials for each outcomes. Table S3. Subgroup analysis results [file 13643_2023_2238_MOESM1_ESM.docx]

**Text S1**

**PubMed(34)**

#1 "Non-alcoholic Fatty Liver Disease"[Mesh]

#2 “Non alcoholic Fatty Liver”[title/abstract] OR “Nonalcoholic Fatty Liver”[title/abstract] OR “Non-alcoholic Fatty Liver”[title/abstract] OR “Non alcoholic Fatty Livers”[title/abstract] OR “Nonalcoholic Fatty Livers”[title/abstract] OR “Non-alcoholic Fatty Livers”[title/abstract] OR NAFLD[title/abstract] OR “Nonalcoholic Steatohepatitis”[title/abstract] OR “Non-alcoholic Steatohepatitis”[title/abstract] OR “Non alcoholic Steatohepatitis”[title/abstract] OR “Nonalcoholic Steatohepatitides” [title/abstract] OR “Non-alcoholic Steatohepatitides” [title/abstract] OR “Non alcoholic Steatohepatitides” [title/abstract]

#3 #1 OR #2

#4 "Fatty Liver"[Mesh]

#5 (liver[title/abstract] OR livers[title/abstract]) AND (fatty[title/abstract] OR steatosis[title/abstract] OR steatoses[title/abstract])

#6 Steatohepatitis[title/abstract] OR Steatohepatitides[title/abstract] OR “Steatosis of liver” [title/abstract] OR “Steatosis of livers” [title/abstract] OR “Steatoses of liver” [title/abstract] OR “Steatoses of livers” [title/abstract] OR “Visceral Steatosis” [title/abstract] OR “Visceral Steatoses” [title/abstract] OR “liver Steatosis” [title/abstract] OR “liver Steatoses” [title/abstract]

#7 #4 OR #5 OR #6

#8 Nonalcoholic[title/abstract] OR Non alcoholic[title/abstract] OR Non-alcoholic[title/abstract]

#9 #7 AND #8

#10 #3 OR #9

#11 "Animals"[Mesh] NOT "Humans"[Mesh]

#12 #10 NOT #11

#13 "Randomized Controlled Trial"[Publication Type] OR "Controlled Clinical Trial"[Publication Type] OR "Clinical Trials as Topic"[Mesh] OR randomized[Title/Abstract] OR randomised[Title/Abstract] OR randomly[Title/Abstract] OR placebo[Title/Abstract] OR trial[Title]

#14 #12 AND #13

#15 "Carnitine"[Mesh]

#16 Carnitine[Title/Abstract] OR Levocarnitine[Title/Abstract] OR “Vitamin BT” [Title/Abstract] OR L-Carnitine[Title/Abstract] OR “L Carnitine” [Title/Abstract] OR Bicarnesine[Title/Abstract] OR “acetyl-l-carnitine” [Title/Abstract] OR “l-acetylcarnitine” [Title/Abstract]

#17 #15 OR #16

#18 #14 AND #17

**Table S1 Inclusion and exclusion criteria of each randomised controlled trials**

| **Author, year** | **Inclusion criteria** | **Exclusion criteria** |
| --- | --- | --- |
| Hossein, 2021 | Patients with NAFLD, increase liver enzymes more than 40 unit/L and diagnosis of fatty liver based on ultrasound, patients between the ages of 5 and 15 years. | Patients with auto immune hepatitis, hypothyroidism, Wilson disease, viral hepatitis, hemochromatosis, gastrointestinal disorders, alcohol consumption, use of drugs that might affect liver functions and participants with low compliance for following the study and failure to complete medication period (3 months). |
| Shirin, 2015(1) | The diagnosis of NASH was done based on ALT levels > 3 times of upper limit of normal range and ultra-sonography outcomes. The including criteria were definite diagnosis of NASH with pericentral macrovascular steatosis, age ranges of 18 to 65 years and BMI>25 kg/m2. | Smoking, pregnancy, use of insulin and blood lipid lowering agents and use of steroid drugs, homochromatosis, consumption of any supplement during the previous month, Wilson’ s disease, use of high doses of estrogen, history of Cushing disease, hyperthyroidism and total peripheral nutrition in the past 6 months were considered as exclusion criteria. |
| Shirin, 2015(2) | The diagnosis of NASH was done based on ALT levels > 3 times of upper limit of normal range and ultra-sonography outcomes. The including criteria were definite diagnosis of NASH with pericentral microvascular steatosis, age ranges of 18 to 65 years and BMI>25 kg/m2. | Smoking, pregnancy, use of insulin and blood lipid lowering agents and use of steroid drugs, homochromatosis, consumption of any supplement during the previous month, Wilson’ s disease, use of high doses of estrogen, history of Cushing disease, hyperthyroidism and total peripheral nutrition in the past 6 months were considered as exclusion criteria. |
| Pezhman, 2016 | Inclusion criteria included known history of diabetes Mellitus type 2, AST and ALT elevation, sonographic findings compatible with fatty liver, absence of any history of viral hepatitis, hemochromatosis, Wilson disease, autoimmune hepatitis, alpha 1 antitrypsin deficiency and or and chronic liver disease and elevated serum TG and cholesterol level. | Exclusion criteria included any history of significant chronic liver disease, history of gastroplasty of jejunoileal bypass surgery, consumption of hepatotoxic medication during last 6 months and significant alcohol consumption (more than 10 g for female and 20 g for male/ day). |
| Bae, 2015 | Individuals aged 20–70 years with known diabetes and NALFD who met all of the following inclusion criteria were eligible to participate in the study: 1) a previous diagnosis of type 2 diabetes at least 3 months before screening, 2) glycated hemoglobin (HbA1c ) greater than 6.4% (46 mmol/mol) or fasting plasma glucose 130–300 mg/dL (inclusive) at screening, and 3) ALT 50–250 IU/L at screening. | Excluded subjects with alcohol intake .30 g/day in men or .20 g/day in women, those with positive serologic markers for hepatitis A, B, or C virus at screening, those with known history or clinical evidence of liver cirrhosis (serum total bilirubin $1.8 mg/dL, albumin ,3.5 g/dL, international normalized ratio .1.3, or platelet count ,150,000/mm3 ), and those with other liver disease such as acute hepatitis or drug-induced hepatitis. Also excluded subjects taking thiazolidinediones for treatment of diabetes, those who received an ant obesity drug within 1 month before screening, those with a history of malignancy, those with a history of severe heart disease (angioplasty, stent placement, bypass surgery, myocardial infarction, unstable angina pectoris, congestive heart failure, or ventricular arrhythmia within 6 months before screening), and women who were pregnant or lactating. |
| Hong, 2014 | Deﬁned NAFLD as a plasma ALT level of 40–250 IU/L in the absence of chronic hepatitis (including hepatitis B or C virus carriers), alcoholic liver diseases, autoimmune liver diseases, or other active liver diseases. | Patients who drank more than three units of alcohol a day twice a week were excluded. Patients who had ever taken insulin or any kind of oral antidiabetic medication were excluded. Women who were pregnant, breastfeeding, or of child-bearing potential without using adequate contraceptive methods were excluded. |
| Mariano, 2010 | Eighty patients with a clinical and [Saneian, 2021 #20] were enrolled in the study. The data included subjects who had, for at least 6 months, abnormal serum aminotransferase levels that were not related to other causes of liver disease | Significant alcohol consumption was a criteria of exclusion ( > 10 g per day for females and > 20 g per day for males). Other causes of exclusion were hereditary hemochromatosis, α-1 antitrypsin deficiency, Wilson’s disease prior surgical procedures such as jejunoileal or jejunocolic bypass, gastroplasty, total parenteral nutrition in the past 6 months, pregnancy, use of drugs such as calcium channel blockers, high dose of synthetic estrogens, methotrexate, amiodarone steroids, chloroquine, a history of treatment with lipid-lowering agents, a history of hypothyroidism, or Cushing syndrome. |
| Mohamad, 2014 | The patient criteria include NAFLD with liver steatoses in sonography of the liver having liver function test with greater than 40mg/dl. | Other reasons increasing liver enzymes: viral infections, hereditary diseases such as hemachromatosis, Wilson`s disease, auto immunity hepatitis and drugs causing drug hepatitis Alcohol consumption more than 10 gr for female and 20 gr for male Sonographic and laboratory evidences confirming cholestatic Diseases triglyceride more than 500 mg/dl in individuals. |

**Table S2 Risk of bias of included randomised controlled trials for each outcomes**

| **Study** | **Outcome** | **Random sequence generation** | **Allocation concealment** | **Blinding of patients** | **Blinding of health care providers** | **Blinding of data collectors** | **Blinding of outcome assessors/ adjudicators** | **Blinding of data analysts** | **Incomplete outcome data** | **Selective outcome reporting** | **Other bias** |
| --- | --- | --- | --- | --- | --- | --- | --- | --- | --- | --- | --- |
| Hossein, 2021 | ALT | Definitely Low | Probably Low | Definitely Low | Definitely Low | Probably High | Probably High | Definitely Low | High | Probably Low | Definitely Low |
| Hossein, 2021 | AST | Definitely Low | Probably Low | Definitely Low | Definitely Low | Probably High | Probably High | Definitely Low | High | Probably Low | Definitely Low |
| Hossein, 2021 | γ-GT | Definitely Low | Probably Low | Definitely Low | Definitely Low | Probably High | Probably High | Definitely Low | High | Probably Low | Definitely Low |
| Hossein, 2021 | BMI | Definitely Low | Probably Low | Definitely Low | Definitely Low | Probably High | Probably High | Definitely Low | High | Probably Low | Definitely Low |
| Hossein, 2021 | Waist circumference | Definitely Low | Probably Low | Definitely Low | Definitely Low | Probably High | Probably High | Definitely Low | High | Probably Low | Definitely Low |
| Hossein, 2021 | Weight | Definitely Low | Probably Low | Definitely Low | Definitely Low | Probably High | Probably High | Definitely Low | High | Probably Low | Definitely Low |
| Hossein, 2021 | Adverse events | Definitely Low | Probably Low | Definitely Low | Definitely Low | Probably High | Probably High | Definitely Low | High | Probably Low | Definitely Low |
| Shirin, 2015(1) | BMI | Probably High | Definitely Low | Definitely Low | Definitely Low | Probably High | Probably High | Probably High | High | Probably Low | Definitely Low |
| Shirin, 2015(1) | Waist circumference | Probably High | Definitely Low | Definitely Low | Definitely Low | Probably High | Probably High | Probably High | High | Probably Low | Definitely Low |
| Shirin, 2015(1) | Weight | Probably High | Definitely Low | Definitely Low | Definitely Low | Probably High | Probably High | Probably High | High | Probably Low | Definitely Low |
| Shirin, 2015(2) | Hs-CRP | Probably High | Definitely Low | Definitely Low | Definitely Low | Probably High | Probably High | Probably High | High | Probably Low | Definitely Low |
| Pezhman, 2016 | AST | Definitely Low | Probably Low | Definitely Low | Definitely Low | Probably High | Probably High | Probably High | High | Probably Low | Definitely Low |
| Pezhman, 2016 | ALT | Definitely Low | Probably Low | Definitely Low | Definitely Low | Probably High | Probably High | Probably High | High | Probably Low | Definitely Low |
| Pezhman, 2016 | Total cholesterol | Definitely Low | Probably Low | Definitely Low | Definitely Low | Probably High | Probably High | Probably High | High | Probably Low | Definitely Low |
| Pezhman, 2016 | Triglyceride | Definitely Low | Probably Low | Definitely Low | Definitely Low | Probably High | Probably High | Probably High | High | Probably Low | Definitely Low |
| Bae, 2015 | ALT | Definitely Low | Definitely Low | Definitely Low | Definitely Low | Probably High | Probably High | Probably High | Low | Probably Low | Definitely Low |
| Bae, 2015 | AST | Definitely Low | Definitely Low | Definitely Low | Definitely Low | Probably High | Probably High | Probably High | Low | Probably Low | Definitely Low |
| Bae, 2015 | γ-GT | Definitely Low | Definitely Low | Definitely Low | Definitely Low | Probably High | Probably High | Probably High | Low | Probably Low | Definitely Low |
| Bae, 2015 | HDL-cholesterol | Definitely Low | Definitely Low | Definitely Low | Definitely Low | Probably High | Probably High | Probably High | Low | Probably Low | Definitely Low |
| Bae, 2015 | LDL-cholesterol | Definitely Low | Definitely Low | Definitely Low | Definitely Low | Probably High | Probably High | Probably High | Low | Probably Low | Definitely Low |
| Bae, 2015 | Triglyceride | Definitely Low | Definitely Low | Definitely Low | Definitely Low | Probably High | Probably High | Probably High | Low | Probably Low | Definitely Low |
| Bae, 2015 | BMI | Definitely Low | Definitely Low | Definitely Low | Definitely Low | Probably High | Probably High | Probably High | Low | Probably Low | Definitely Low |
| Bae, 2015 | Waist circumference | Definitely Low | Definitely Low | Definitely Low | Definitely Low | Probably High | Probably High | Probably High | Low | Probably Low | Definitely Low |
| Bae, 2015 | Weight | Definitely Low | Definitely Low | Definitely Low | Definitely Low | Probably High | Probably High | Probably High | Low | Probably Low | Definitely Low |
| Bae, 2015 | Adverse events | Definitely Low | Definitely Low | Definitely Low | Definitely Low | Probably High | Probably High | Probably High | Low | Probably Low | Definitely Low |
| Hong, 2014 | ALT | Probably Low | Probably Low | Definitely Low | Definitely Low | Probably High | Probably High | Probably High | Low | Probably Low | Definitely Low |
| Hong, 2014 | AST | Probably Low | Probably Low | Definitely Low | Definitely Low | Probably High | Probably High | Probably High | Low | Probably Low | Definitely Low |
| Hong, 2014 | γ-GT | Probably Low | Probably Low | Definitely Low | Definitely Low | Probably High | Probably High | Probably High | Low | Probably Low | Definitely Low |
| Hong, 2014 | HDL-cholesterol | Probably Low | Probably Low | Definitely Low | Definitely Low | Probably High | Probably High | Probably High | Low | Probably Low | Definitely Low |
| Hong, 2014 | LDL-cholesterol | Probably Low | Probably Low | Definitely Low | Definitely Low | Probably High | Probably High | Probably High | Low | Probably Low | Definitely Low |
| Hong, 2014 | Total cholesterol | Probably Low | Probably Low | Definitely Low | Definitely Low | Probably High | Probably High | Probably High | Low | Probably Low | Definitely Low |
| Hong, 2014 | Triglyceride | Probably Low | Probably Low | Definitely Low | Definitely Low | Probably High | Probably High | Probably High | Low | Probably Low | Definitely Low |
| Hong, 2014 | BMI | Probably Low | Probably Low | Definitely Low | Definitely Low | Probably High | Probably High | Probably High | Low | Probably Low | Definitely Low |
| Hong, 2014 | hs-CRP | Probably Low | Probably Low | Definitely Low | Definitely Low | Probably High | Probably High | Probably High | Low | Probably Low | Definitely Low |
| Hong, 2014 | Adverse events | Probably Low | Probably Low | Definitely Low | Definitely Low | Probably High | Probably High | Probably High | Low | Probably Low | Definitely Low |
| Mariano, 2010 | ALT | Definitely Low | Probably Low | Definitely Low | Definitely Low | Probably High | Probably High | Probably High | Low | Probably Low | Definitely Low |
| Mariano, 2010 | AST | Definitely Low | Probably Low | Definitely Low | Definitely Low | Probably High | Probably High | Probably High | Low | Probably Low | Definitely Low |
| Mariano, 2010 | γ-GT | Definitely Low | Probably Low | Definitely Low | Definitely Low | Probably High | Probably High | Probably High | Low | Probably Low | Definitely Low |
| Mariano, 2010 | HDL-cholesterol | Definitely Low | Probably Low | Definitely Low | Definitely Low | Probably High | Probably High | Probably High | Low | Probably Low | Definitely Low |
| Mariano, 2010 | LDL-cholesterol | Definitely Low | Probably Low | Definitely Low | Definitely Low | Probably High | Probably High | Probably High | Low | Probably Low | Definitely Low |
| Mariano, 2010 | Total cholesterol | Definitely Low | Probably Low | Definitely Low | Definitely Low | Probably High | Probably High | Probably High | Low | Probably Low | Definitely Low |
| Mariano, 2010 | Triglyceride | Definitely Low | Probably Low | Definitely Low | Definitely Low | Probably High | Probably High | Probably High | Low | Probably Low | Definitely Low |
| Mariano, 2010 | BMI | Definitely Low | Probably Low | Definitely Low | Definitely Low | Probably High | Probably High | Probably High | Low | Probably Low | Definitely Low |
| Mohamad, 2014 | AST | Probably Low | Probably Low | Probably Low | Probably Low | Probably Low | Probably Low | Probably Low | Low | Probably Low | Definitely Low |
| Mohamad, 2014 | ALT | Probably Low | Probably Low | Probably Low | Probably Low | Probably Low | Probably Low | Probably Low | Low | Probably Low | Definitely Low |
| Mohamad, 2014 | BMI | Probably Low | Probably Low | Probably Low | Probably Low | Probably Low | Probably Low | Probably Low | Low | Probably Low | Definitely Low |
| Mohamad, 2014 | Weight | Probably Low | Probably Low | Probably Low | Probably Low | Probably Low | Probably Low | Probably Low | Low | Probably Low | Definitely Low |

① Liver function tests(AST, ALT, r-GT); ② Lipid profile tests(HDL-cholesterol, LDL-cholesterol, Total cholesterol, Triglyceride); ③ Body Indicators(BMI, Weight, Waist circumference); ④ Inflammatory factors(CRP); ⑤ Adverse Events

**Table S3 Subgroup analysis results**

| **Outcomes** | **Subgroups** | **No. studies** | **No. sample sizes** | **MD** | **95%CI** | **P_within group** | **Certainty of evidence** | **Results of ICEMAN** |
| --- | --- | --- | --- | --- | --- | --- | --- | --- |
| ①Changes in the AST level | Health status_NAFLD | 5 | 332 | -13.9 | (-28.97, 1.17) | 0.18 | Low | -- |
|  | Health status_NASH | 1 | 74 | -25.6 | (-33.63, -17.57) |  |  |  |
|  | Age_younger | 1 | 62 | 0.5 | (-0.70, 1.70) | **< 0.01** |  | Moderate credibility |
|  | Age_adult | 5 | 344 | -20.3 | (-28.62, -12.28) |  |  |  |
|  | Daily dose_≥ 1000mg | 3 | 196 | -17.2 | (-38.84, 4.39) | 0.84 |  | -- |
|  | Daily dose_< 1000mg | 3 | 210 | -14.6 | (-27.30, -1.89) |  |  |  |
|  | Duration_12 weeks | 4 | 252 | -13.7 | (-31.27, 3.97) | 0.46 |  | -- |
|  | Duration_24 weeks | 2 | 154 | -21.3 | (-31.53, -11.15) |  |  |  |
| ①Changes in the ALT level | Health status_NAFLD | 5 | 332 | -27.7 | (-51.03, -4.32) | 0.59 | Low | -- |
|  | Health status_NASH | 1 | 74 | -21.0 | (-27.76, -14.24) |  |  |  |
|  | Age_younger | 1 | 62 | 0.4 | (-1.32, 2.12) | **< 0.01** |  | Moderate credibility |
|  | Age_adult | 5 | 344 | -31.7 | (-47.61, -15.79) |  |  |  |
|  | Daily dose_≥ 1000mg | 3 | 196 | -19.1 | (-44.94, 6.75) | 0.52 |  | -- |
|  | Daily dose_< 1000mg | 3 | 210 | -34.8 | (-74.80, 5.16) |  |  |  |
|  | Duration_12 weeks | 4 | 252 | -34.2 | (-62.41, -5.93) | 0.20 |  | -- |
|  | Duration_24 weeks | 2 | 154 | -12.2 | (-30.34, 5.87) |  |  |  |
| ①Changes in the γ-GT level | Health status_NAFLD | 2 | 130 | -3.6 | (-26.54, 19.27) | 0.27 | Low | -- |
|  | Health status_NASH | 1 | 74 | -17.2 | (-25.23, -9.17) |  |  |  |
|  | Daily dose_≥ 1000mg | 2 | 130 | -3.6 | (-26.54, 19.27) | 0.27 |  | -- |
|  | Daily dose_< 1000mg | 2 | 74 | -17.2 | (-25.23, -9.17) |  |  |  |
|  | Duration_12 weeks | 2 | 130 | -3.6 | (-26.54, 19.27) | 0.27 |  | -- |
|  | Duration_24 weeks | 1 | 74 | -17.2 | (-25.23, -9.17) |  |  |  |
| ②Changes in the HDL-cholesterol | Health status_NAFLD | 2 | 130 | 0.0 | (-2.04, 2.04) | 0.22 | Moderate | -- |
|  | Health status_NASH | 1 | 74 | 1.4 | (0.39, 2.49) |  |  |  |
|  | Daily dose_≥ 1000mg | 2 | 130 | 0.0 | (-2.04, 2.04) | 0.22 |  | -- |
|  | Daily dose_< 1000mg | 1 | 74 | 1.4 | (0.39, 2.49) |  |  |  |
|  | Duration_12 weeks | 2 | 130 | 0.0 | (-2.04, 2.04) | 0.22 |  | -- |
|  | Duration_24 weeks | 1 | 74 | 1.44 | (0.39, 2.49) |  |  |  |
| ②Changes in the LDL-cholesterol | Health status_NAFLD | 2 | 130 | 1.7 | (-4.66, 8.06) | **< 0.01** | Low | Low credibility |
|  | Health status_NASH | 1 | 74 | -20.9 | (-27.92, -13.84) |  |  |  |
|  | Daily dose_≥ 1000mg | 2 | 130 | 1.7 | (-4.66, 8.06) | **< 0.01** |  | Low credibility |
|  | Daily dose_< 1000mg | 1 | 74 | -20.9 | (-27.92, -13.84) |  |  |  |
|  | Duration_12 weeks | 2 | 130 | 1.7 | (-4.66, 8.06) | **< 0.01** |  | Low credibility |
|  | Duration_24 weeks | 1 | 74 | -20.9 | (-27.92, -13.84) |  |  |  |
| ②Changes in the Total cholesterol | Health status_NAFLD | 2 | 112 | -2.5 | (-14.85, 9.81) | **0.01** | Low | Low credibility |
|  | Health status_NASH | 1 | 74 | -20.7 | (-28.22, -13.22) |  |  |  |
|  | Daily dose_≥ 1000mg | 2 | 134 | -20.3 | (-27.68, -12.92) | **0.02** |  | Low credibility |
|  | Daily dose_< 1000mg | 1 | 52 | -2.1 | (-15.00, 10.80) |  |  |  |
|  | Duration_12 weeks | 2 | 112 | -2.5 | (-14.85, 9.81) | **0.01** |  | Low credibility |
|  | Duration_24 weeks | 1 | 74 | -20.7 | (-28.22, -13.22) |  |  |  |
| ②Changes in the Triglyceride | Health status_NAFLD | 3 | 160 | -16.4 | (-56.44, 23.57) | 0.64 | Moderate | -- |
|  | Health status_NASH | 1 | 74 | -6.6 | (-13.72, 0.48) |  |  |  |
|  | Daily dose_≥ 1000mg | 3 | 160 | -16.4 | (-56.44, 23.57) | 0.64 |  | -- |
|  | Daily dose_< 1000mg | 1 | 74 | -6.6 | (-13.72, 0.48) |  |  |  |
|  | Duration_12 weeks | 3 | 160 | -16.4 | (-56.44, 23.57) | 0.64 |  | -- |
|  | Duration_24 weeks | 1 | 74 | -6.6 | (-13.72, 0.48) |  |  |  |
| ③Changes in the BMI | Health status_NAFLD | 5 | 343 | 0.01 | (-0.24, 0.27) | 0.81 | Moderate | -- |
|  | Health status_NASH | 1 | 74 | -0.2 | (-1.91, 1.51) |  |  |  |
|  | Age_younger | 1 | 62 | -0.2 | (-0.30, -0.10) | **< 0.01** |  | Moderate credibility |
|  | Age_adult | 5 | 355 | 0.1 | (-0.07, 0.31) |  |  |  |
|  | Daily dose_≥ 1000mg | 3 | 207 | 0.1 | (-0.57, 0.68) | 0.90 |  | -- |
|  | Daily dose_< 1000mg | 3 | 210 | 0.1 | (-0.10, 0.29) |  |  |  |
|  | Duration_12 weeks | 4 | 263 | 0.03 | (-0.23, 0.30) | 0.46 |  | -- |
|  | Duration_24 weeks | 2 | 154 | -0.4 | (-1.58, 0.75) |  |  |  |
| ③Changes in the Weight | Age_younger | 1 | 62 | -0.3 | (-0.53, -0.07) | 0.13 | Moderate | -- |
|  | Age_adult | 3 | 229 | 0.23 | (-0.41, 0.87) |  |  |  |
|  | Daily dose_≥ 1000mg | 2 | 133 | -0.3 | (-0.52, -0.08) | 0.11 |  | -- |
|  | Daily dose_< 1000mg | 2 | 158 | 0.3 | (-0.40, 0.92) |  |  |  |
|  | Duration_12 weeks | 3 | 211 | -0.1 | (-0.55, 0.27) | 0.48 |  | -- |
|  | Duration_24 weeks | 1 | 80 | -2.0 | (-7.15, 3.15) |  |  |  |
| ③Changes in the Waist circumference | Age_younger | 1 | 62 | -0.3 | (-0.57, -0.07) | 0.78 | Low | -- |
|  | Age_adult | 2 | 149 | -0.7 | (-3.46, 2.03) |  |  |  |
|  | Duration_12 weeks | 2 | 133 | -1.1 | (-2.85, 0.62) | 0.1 |  | -- |
|  | Duration_24 weeks | 1 | 78 | 0.7 | (-0.63, 2.03) |  |  |  |
| ④Changes in the hs_CRP | Daily dose_≥ 1000mg | 1 | 71 | -2.19 | (-2.98, -1.40) | **< 0.01** | Very low | Very low credibility |
|  | Daily dose_< 1000mg | 1 | 52 | 0.05 | (-0.07, 0.17) |  |  |  |

① Liver function tests(AST, ALT, r-GT); ② Lipid profile tests(HDL-cholesterol, LDL-cholesterol, Total cholesterol, Triglyceride); ③ Body Indicators(BMI, Weight, Waist circumference); ④ Inflammatory factors(CRP); ⑤ Adverse Events
